# Antiprotozoal Potential of Cultivated *Geranium macrorrhizum* Against *Giardia duodenalis*, *Trichomonas gallinae* and *Leishmania infantum*

**DOI:** 10.3390/ijms27021125

**Published:** 2026-01-22

**Authors:** Sara Marcos-Herraiz, María José Irisarri-Gutiérrez, Javier Carrión, Iris Azami Conesa, Rodrigo Suárez Lombao, Juliana Navarro-Rocha, Jose Francisco Quilez del Moral, Alejandro Fernández Barrero, Eneko Ochoa Larrigan, Azucena González-Coloma, María Teresa Gómez-Muñoz, María Bailén

**Affiliations:** 1 Department of Preventive Medicine, Public Health and Microbiology, Faculty of Medicine, Universidad Autónoma de Madrid, 28029 Madrid, Spain; sara.marcos@uam.es (S.M.-H.); maria.irisarri@uam.es (M.J.I.-G.); 2 ICPVet Research Group, Department of Animal Health, Faculty of Veterinary Medicine, Complutense University of Madrid, 28040 Madrid, Spain; javier.carrion@ucm.es (J.C.); rodrsuar@ucm.es (R.S.L.); mariateg@ucm.es (M.T.G.-M.); 3 Research Institute Hospital 12 de Octubre, 28041 Madrid, Spain; 4 Department of Pharmacy and Nutrition, Faculty of Biomedical and Health Sciences, Universidad Europea de Madrid, 28670 Madrid, Spain; iris.azami@universidadeuropea.es; 5 Department of Plant Science, Agrifood Research and Technology Centre of Aragon (CITA), Avda. Montañana 930, 50059 Zaragoza, Spain; jnavarroro@cita-aragon.es; 6 Agrifood Institute of Aragon—IA2 (Calle Miguel Servet, 177), University of Zaragoza, 50013 Zaragoza, Spain; 7 Department of Organic Chemistry, Institute of Biotechnology, University of Granada, 18071 Granada, Spain; jfquilez@ugr.es (J.F.Q.d.M.); afbarre@ugr.es (A.F.B.); 8 Aleovitro S.L., Pol. Pinoa 8, 48170 Zamudio, Spain; eochoa@aleovitro.com; 9 Instituto de Ciencias Agrarias, Consejo Superior de Investigaciones Científicas, 28006 Madrid, Spain; azu@ica.csic.es

**Keywords:** *Trichomonas*, *Giardia*, *Leishmania*, germacrone, natural products, essential oils

## Abstract

Plant-derived natural products are an invaluable source of structurally diverse secondary metabolites with ecological and pharmacological significance. *Geranium macrorrhizum*, a species known for producing essential oils rich in monoterpenoids and sesquiterpenes, has been scarcely explored for its antiparasitic potential. This study represents the first comprehensive evaluation of the antiprotozoal activity of *G. macrorrhizum* obtained from cultivated plants. Plant material was produced under controlled greenhouse cultivation systems, ensuring high-quality and reproducible metabolite profiles. Essential oils were obtained through hydrodistillation and chemically characterized by Gas Chromatography-Mass Spectrometry (GC–MS). In vitro assays were conducted against *Giardia duodenalis*, *Trichomonas gallinae*, and *Leishmania infantum* to assess antiparasitic efficacy and cytotoxicity. The results demonstrated strong activity of essential oils against *Trichomonas gallinae*, and *Leishmania infantum*, indicating the relevance of lipophilic compounds—especially germacrone—as key bioactive constituents. Germacrone exhibited strong and selective antiparasitic activity, outperforming its structural analogues. Microscopic analyses revealed distinct parasite-specific morphological alterations, differing from those induced by conventional drugs such as metronidazole and amphotericin B. These findings highlight *G. macrorrhizum* obtained through biotechnological cultivation as a novel and sustainable source of natural antiprotozoal agents. The study underscores the importance of integrating controlled cultivation with phytochemical and biological evaluation to advance the discovery of innovative bioactive compounds.

## 1. Introduction

Protozoan pathogens, *Giardia duodenalis* (Stiles, 1902) (*G. duodenalis*), *Leishmania* spp. (Ross, 1903), and *Trichomonas* spp. (Neumann, 1899), represent serious health threats to humans, livestock, and wildlife, and the efficacy of conventional treatments such as nitroimidazoles, amphotericin B, and antimonial compounds is waning due to rising drug resistance [1,2,3,4]. *Leishmania* spp. cause leishmaniasis, a group of diseases transmitted through the bite of infected female phlebotomine sandflies. More than one billion people currently live in areas endemic for leishmaniasis and are at risk of infection [4]. Treatment primarily relies on pharmacological therapies; however, many available drugs (pentavalent antimonials, liposomal amphotericin B, and pentamidine) are associated with high toxicity, limited efficacy, complex administration protocols, or significant costs. Furthermore, resistant strains have emerged, complicating disease management [3]. *G. duodenalis* is an extracellular enteric protozoan responsible for widespread diarrheal diseases, with over 300 million cases reported annually, particularly in low-income and developing regions [5]. Despite the availability of treatments, the increasing resistance of *Giardia* to nitroimidazole-based therapies, such as metronidazole (MTZ), poses a growing concern [1,6]. In birds, *Trichomonas gallinae* ((Rivolta) Stabler 1938) (*T. gallinae*), is a flagellated oropharyngeal parasite that causes granulomas and starvation, significantly impacting avian populations. While Columbiformes serve as the primary reservoirs, other domestic and wild bird species are also susceptible [7]. The current treatment for *T. gallinae* infections also relies on nitroimidazoles, yet no preventive treatments have been approved in the EU, and therapy failures linked to resistant strains have been documented [8]. There is an urgent need for alternative treatments against *Leishmania* spp., *G. duodenalis*, and *Trichomonas* spp.

In this context, medicinal plants emerge as a vital reservoir of novel anti-protozoal agents. Their rich repertoire of secondary metabolites, honed through millennia of ecological interactions and stress-response mechanisms [9], provides an unparalleled source of chemically diverse scaffolds. These phytochemicals—alkaloids, terpenoids, flavonoids, and phenolics—exhibit a broad spectrum of bioactivities, including direct antiparasitic effects, modulation of host immune pathways, and synergistic enhancement of existing drugs. Consequently, the structural diversity and potent biological activity of plant-derived natural products remain central to contemporary efforts to discover and develop next-generation therapeutics against resistant protozoan infections [10]. Therefore, medicinal plants remain important in modern drug discovery due to their rich source of diverse secondary metabolites, the foundation of traditional knowledge, and the advancement of modern technology for research and development [11].

*Geranium macrorrhizum* L. (1753), also known by its common names bigroot geranium, Bulgarian geranium, and rock crane’s-bill, is a perennial aromatic species within Geraniaceae, found across temperate regions of the Northern Hemisphere [12]. *G. macrorrhizum* essential oil (EO), rich in monoterpenoids (e.g., geraniol, β-citronellol) and sesquiterpenes (notably germacrone), showed insecticidal, acaricidal, antifungal, and antibacterial effects [13,14]. As a medicinal plant, *G. macrorrhizum* extracts and EOs have antimicrobial, antioxidant, hypotensive, spasmolytic, astringent, and sedative properties [14,15,16]. However, the antiprotozoal potential of *G. macrorrhizum* remains untested despite extensive studies on other *Geranium* species (e.g., *Geranium mexicanum* Kunth, (1822), *Geranium wallichianum* D. Don ex Sweet, (1821)) with antiprotozoal activity (against *Giardia*, *Leishmania*, and *Trichomonas* spp.) [17,18,19].

A previous study demonstrated marked differences in terpene composition between wild and commercial-ornamental *G. macrorrhizum* populations: wild plants were enriched in β-elemenone, thymol, and germacrone, whereas commercial cultivars were dominated by linalool and linalyl acetate, with respective insecticidal and acaricidal activity differences. This emphasizes how cultivation, genotype, and growing conditions shape phytochemical profiles and bioactivity [13]. In this context, controlled cultivation of medicinal plants in greenhouse environments offers a route to produce consistent plant biomass, potentially mitigating environmental variation and optimizing metabolite production [20].

In this study, we evaluate for the first time the antiprotozoal potential against *G. duodenalis*, *T. gallinae*, and *Leishmania infantum* (Nicolle, 1908) (*L. infantum*) of essential oils from a greenhouse produced *G. macrorrhizum* selected cultivar [13]. Furthermore, the main components of the essential oils and their semi-synthetic derivatives have also been tested along with their cytotoxicity to establish their selective action.

## 2. Results

### 2.1. Antiparasitic Activity of Essential Oils from G. macrorrhizum

EOs from *G. macrorrhizum* were evaluated for their antiparasitic activity against *T. gallinae* and *G. duodenalis* trophozoites, as well as *L. infantum* promastigotes (Table 1). EOs from greenhouse flowers (EOF) demonstrated greater activity than those from aerial parts (EOAP) for *G. duodenalis* and *T. gallinae*. However, only EOAP showed activity against *L. infantum* (IC_50_ = 69.6 μg/mL). Overall, EOAP was the most effective against *L. infantum*, while EOF demonstrated the highest activity against *T. gallinae*.

### 2.2. Chemical Composition of G. macrorrhizum EOs

The chemical composition of EOs from *G. macrorrhizum* aerial parts and flowers was determined by GC-MS (Table 2). β-Elemenone and germacrone were the main constituents of the essential oils (EOF and EOAP). β-Elemenone was more abundant in flowers (EOF), while germacrone predominated in aerial parts (EOAP).

### 2.3. Antiparasitic and Cytotoxic Properties of G. macrorrhizum Main Compounds Against Extracellular Protozoa

**Figure 1:**
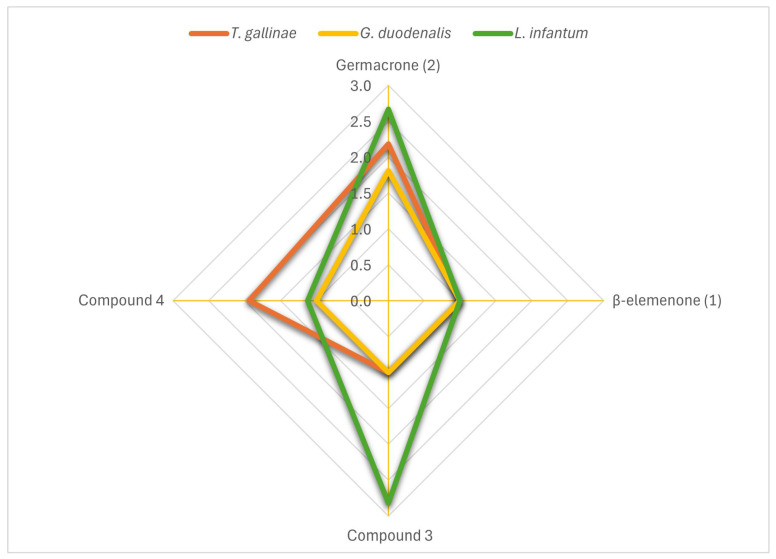
Selectivity indexes of main components of. *G. macrorrhizum* on extracellular protozoa (*T. gallinae* and *G. duodenalis* trophozoites and *L. infantum* promastigotes).

β -Elemenone- (**1**), germacrone- (**2**) and germacrone-derived compounds, **3** and **4**, were also tested (Table 3). Metronidazole was used as the reference compound for anti-*Giardia* and anti-*Trichomonas* activity, while Amphotericin B served as the reference for anti-*Leishmania* activity. Among them, germacrone was effective against all three protozoa (*L. infantum*, *G. duodenalis*, and *T. gallinae*), whereas β-elemenone showed no activity. Compound **3** exhibited activity exclusively against *L. infantum*, while **4** was effective against *T. gallinae* and displayed moderate activity against *L. infantum*. In summary, *L. infantum* was the most sensitive parasite and germacrone (**2**) the compound with the broader spectrum of action (Figure 1).

### 2.4. Antileishmanial Effects of Germacrone on Intracellular Amastigotes

**Figure 2:**
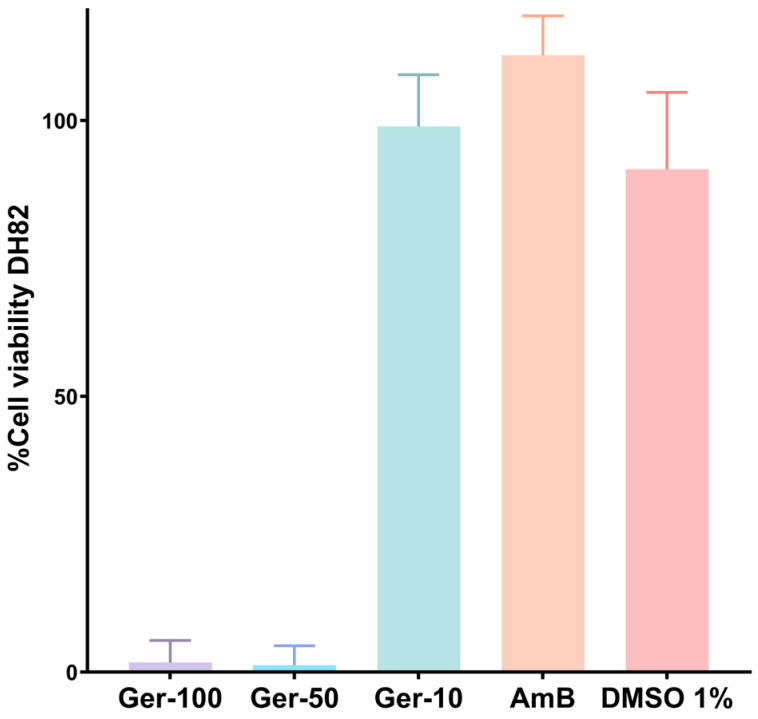
Cytotoxic effects of germacrone in DH82 canine macrophages expressed as % cell viability (Ger-100: germacrone 100 µg/mL; Ger-50: germacrone 50 µg/mL; Ger-10: germacrone 10 µg/mL; AmB: amphotericin B 1 µg/mL); solvent DMSO 1%; Data from two independent experiments (biological replicates) in quadruplicates (technical replicates) are shown. CC_50_ value was calculated from dose–response curve using viability assays.

The antileishmanial effects of germacrone (**2**) were also evaluated on intracellular amastigotes in DH82 canine macrophages (Table 4). Prior to this, the cytotoxicity of germacrone was assessed in DH82 macrophages, yielding a CC_50_ value of 12.9 (11.4–14.6) (R^2^: 98.4) μg/mL (Figure 2). At concentrations of 50 and 100 μg/mL, germacrone was lethal to canine macrophages. When evaluating the effect of germacrone (**2**) on amastigote-infected canine macrophages, the percentage of infected cells decreased from 42.8% (control) to 25.9%, in 10 µg/mL germacrone, resulting in a reduction of the infection index by more than 50% (from 152.1 to 61.0).

### 2.5. Morphological Evaluation of the Anti-Protozoan Effects of Germacrone

**Figure 3:**
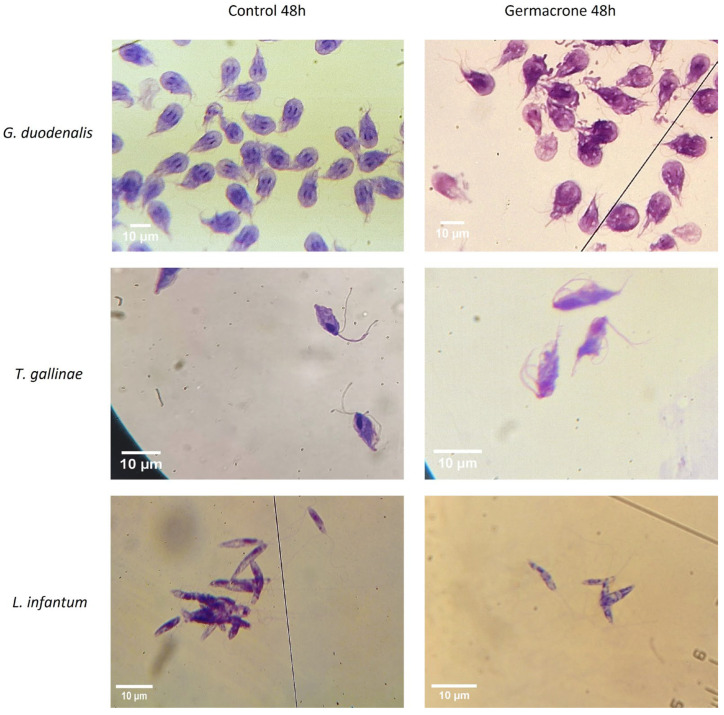
Effect of germacrone (100 µg/mL) on *G. duodenalis* and *T. gallinae* trophozoites and *L. infantum* promastigotes stained with Diff-Quik after 48 h of exposure observed under light microscopy (×40 *G. duodenalis*; ×100 *T. gallinae* and *L. infantum*).

The antiprotozoal effects of germacrone on *G. duodenalis* and *T. gallinae* trophozoites and *L. infantum* promastigotes at 48 h of exposure were further analyzed by light microscopy to detect morphological changes (Figure 3). In *G. duodenalis* trophozoites, germacrone provoked a less defined cytoplasm and nucleus of the cells. In *T. gallinae* trophozoites, an elongation of the cytoplasm and the nucleus could be observed. The effect on *L. infantum* promastigotes was less pronounced.

#### 2.5.1. *Giardia duodenalis*

**Figure 4:**
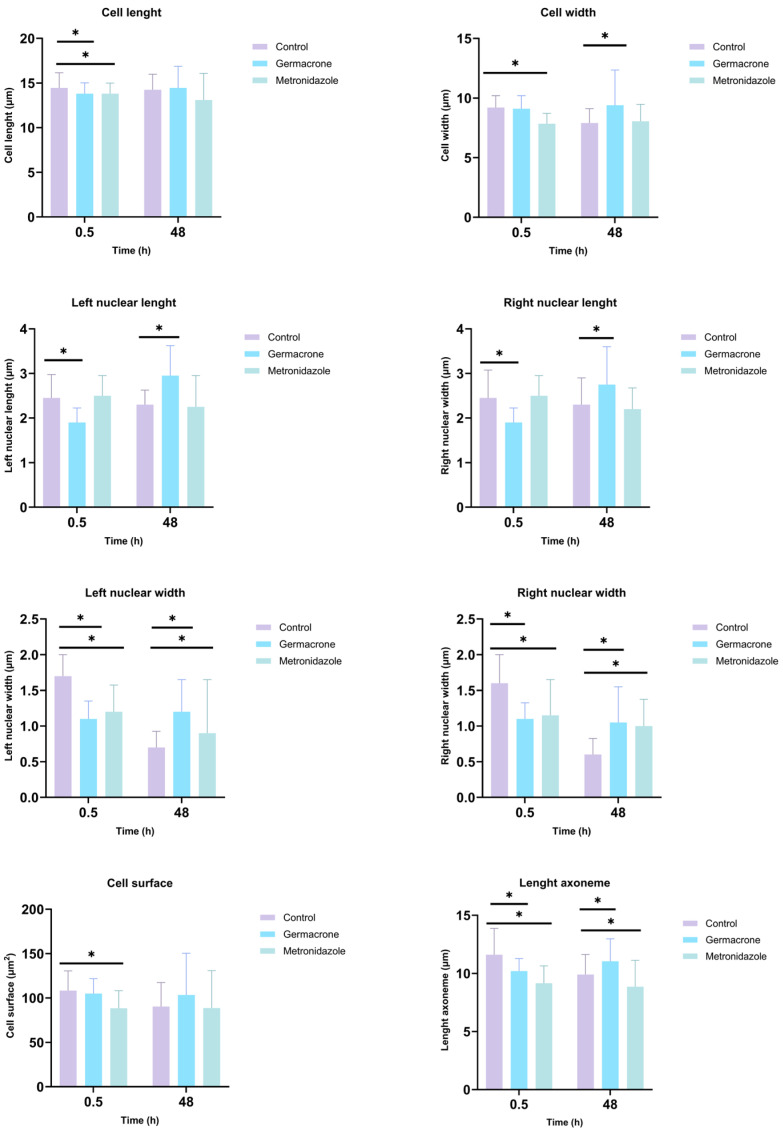
Effect of germacrone (100 µg/mL) and metronidazole (100 µg/mL) on *G. duodenalis* morphology (cell length, cell width, cell surface, length of axoneme, nuclear length and nuclear width) after 0.5 and 48 h of exposure (* *p* < 0.05, Mann–Whitney U test); *n* = 30 technical replicates; control: DMSO 1%; solvent: DMSO 1%.

The effects of germacrone and metronidazole on various cellular parameters on *G. duodenalis* were analyzed throughout the study (Figure 4). For cell length, both germacrone and metronidazole showed significant reductions at 30 min of exposure compared to the control group. Regarding cell width, no differences were observed at 0.5 h with germacrone, but after 48 h, a significant increase in cell width was noted. In contrast, metronidazole led to a decrease in cell width at 0.5 h when compared to the control. For nuclear length, both the left and right nuclear lengths decreased significantly with germacrone at 0.5 h and later increased after 48 h, relative to the control. No effects were observed for metronidazole on nuclear length. Regarding nuclear width, both germacrone and metronidazole caused a reduction at 0.5 h, followed by an increase at 48 h when compared to the control. The axoneme length decreased at 0.5 h for both germacrone and metronidazole; however, at 48 h, germacrone caused an increase, while metronidazole continued to decrease compared to the control. Finally, only metronidazole induced a reduction in cell surface area relative to the control within 0.5 h of exposure.

#### 2.5.2. *Trichomonas gallinae*

**Figure 5:**
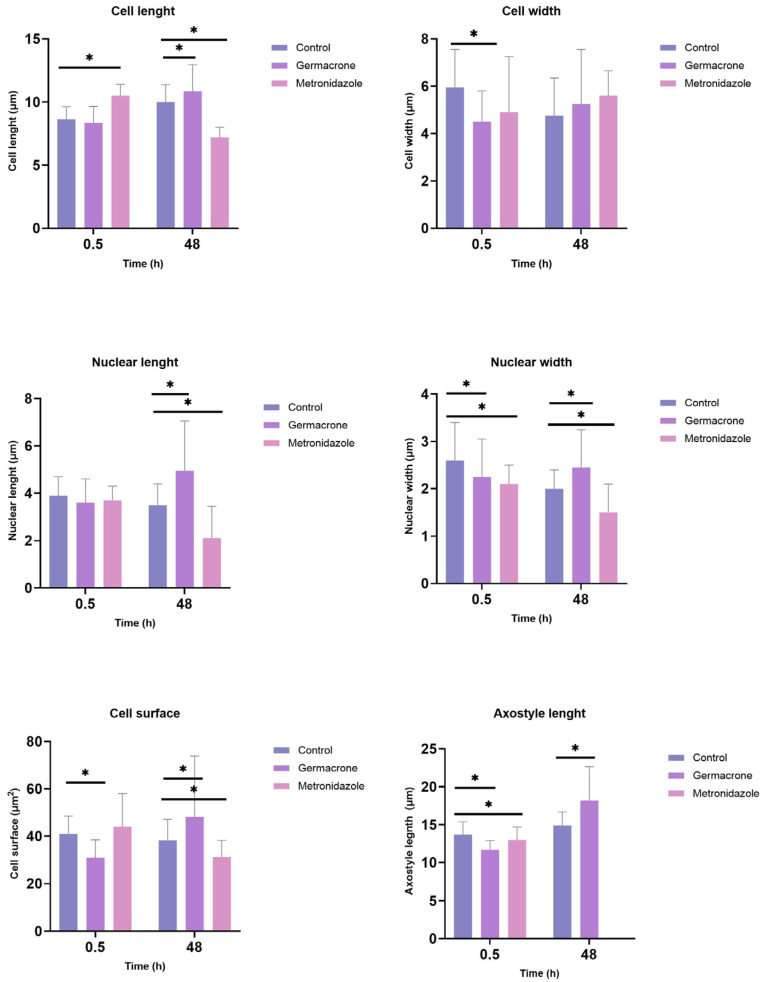
Effect of germacrone (100 µg/mL) and metronidazole (10 µg/mL) on *T. gallinae* morphology (cell length, cell width, cell surface, length of axostyle, nuclear length and nuclear width) after 0.5 and 48 h of exposure (* *p* < 0.05, Mann–Whitney U test); *n* = 30 technical replicates; control: DMSO 1%; solvent: DMSO 1%.

Optical microscopy analysis of *T. gallinae* trophozoites revealed significant morphological alterations following treatment with germacrone and metronidazole (Figure 5). Regarding cell length, germacrone induced an increase after 48 h, whereas metronidazole caused an initial increase at 0.5 h, followed by a reduction at 48 h. Cell width exhibited a significant decrease exclusively in germacrone-treated parasites compared to the control at 0.5 h of exposure. Nuclear length increased upon germacrone exposure but decreased with metronidazole, whereas nuclear width diminished at 0.5 h for both compounds. By 48 h, nuclear width increased in germacrone-treated trophozoites but further decreased in those exposed to metronidazole. Cell surface area decreased transiently at 0.5 h but increased at 48 h with germacrone, whereas metronidazole induced a significant reduction only at 48 h. Axostyle length decreased at 0.5 h for both treatments; however, after 48 h, it increased in germacrone-exposed parasites. Notably, the axostyle became undetectable in most metronidazole-treated trophozoites by 48 h.

#### 2.5.3. *Leishmania infantum*

**Figure 6:**
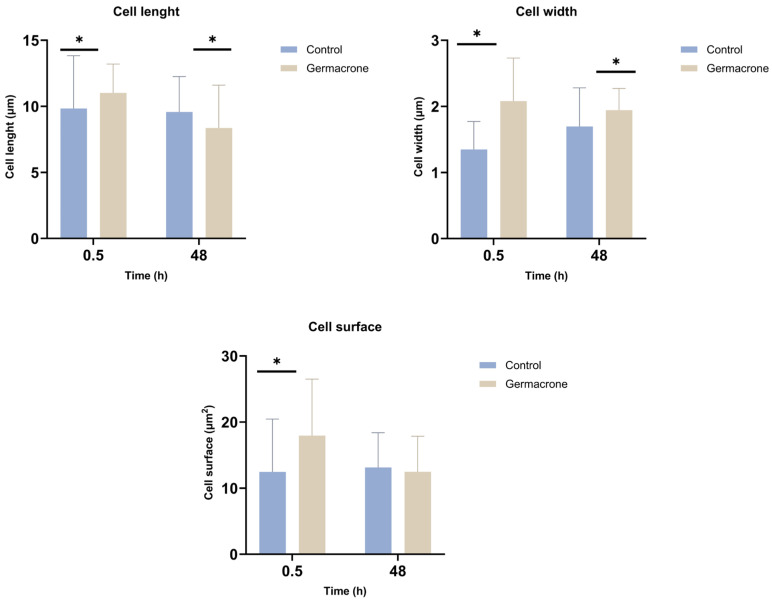
Effect of germacrone (100 µg/mL) on *L. infantum* promastigotes morphology (cell length, cell width and cell surface after 0.5 and 48 h of exposure (* *p* < 0.05, Mann–Whitney U test); *n* = 30 technical replicates; control: DMSO 1%; solvent: DMSO 1%.

Optical microscopy analysis of *L. infantum* promastigotes revealed time-dependent morphological changes following germacrone exposure at 0.5 and 48 h (Figure 6). Cell length exhibited an initial increase at 0.5 h but subsequently decreased at 48 h compared to the control. In contrast, cell width increased significantly at both 0.5 and 48 h post-treatment. Cell surface area transiently increased at 0.5 h with no significant changes detected at 48 h relative to the control. Amphotericin B treated promastigotes suffered strong morphological alterations that impede taking measures of the structures.

## 3. Discussion

The genus *Geranium* L. (eng. Cranesbills) comprises approximately 250 species of herbaceous plants that inhabits the Norther Hemisphere and tropical mountainous regions [24]. Despite demonstrating antimicrobial, insecticidal, ixodicidal, antioxidant, hypotensive, and sedative properties, the antiprotozoal potential of *G. macrorrhizum* EOs remains uninvestigated [13,14,15,16].

In the present study, the evaluation of EOs from *G. macrorrhizum* revealed broad-spectrum activity against *T. gallinae*, *G. duodenalis*, and *L. infantum*. β-Elemenone and germacrone were identified as the major constituents of the analyzed EOs from aerial parts and flowers.

Previous reports of antiprotozoal activity in related *Geranium* species, are related to organic extracts, including methanolic root extracts of *Geranium niveum* (S. Watson, 1886) active against *G. duodenalis* (IC_50_ = 20.64 µg/mL) and *Entamoeba histolytica* (Schaudinn, 1903) (IC_50_ = 8.70 µg/mL) [25]; *G. mexicanum* methanolic extract active against *Trichomonas vaginalis* (Donné, 1836) (IC_50_ = 56.0 µg/mL) [19], while its dichloromethane–methanol root extracts showed moderate efficacy against *G. duodenalis* (IC_50_ = 100.4 µg/mL) [18]; ethanolic and methanolic extracts of *Geranium thunbergii* Siebold & Zucc. ex Lindl. & Paxton (1851), showed potent antimalarial activity [26], and leaf extracts of *G. wallichianum* demonstrated antileishmanial effects against both amastigote and promastigote forms of *Leishmania tropica* (Wright, 1903) [17]. However, this is the first report on the antiparasitic effects of *G. macrorrhizum* essential oil. Collectively, these studies highlight the broad antiparasitic potential of *Geranium* species, supporting the ethnopharmacological relevance of this genus.

Several EOs containing germacrone have demonstrated antiparasitic activity, highlighting the relevance of this sesquiterpene in natural product-based therapeutics. The EO of *Psidium brownianum* ex DC. (1828), which contains approximately 16% germacrone, exhibited significant activity against promastigotes of *Leishmania braziliensis* (Vianna, 1911) and *L. infantum*, with IC_50_ values of 37.53 µg/mL and 75.83 µg/mL, respectively [27]. Similarly, the EO of *Eugenia uniflora* L. (1753), containing 8.52% germacrone, showed inhibitory effects on *Leishmania amazonensis* (Lainson & Shaw, 1972) promastigotes [28]. In addition to germacrone, other sesquiterpenes have been reported to possess antiparasitic properties. For instance, γ-elemene and curzerene have shown activity against both amastigote and promastigote forms of *L. amazonensis* [29]. Other sesquiterpenes (euparin and santhemoidin C) or sequiterpene lactones (anhydroartemorin, cis,trans-costunolide-14-acetate, and 4-hydroxyarbusculin A) demonstrated efficacy against *L. infantum* [30], *L. amazonensis*, and *L. donovani* (Laveran et Mesnil, 1903) [31], respectively. The antiparasitic mechanisms of sesquiterpenes against *Leishmania* spp. are thought to involve the induction of apoptosis, necrosis, autophagy, and disruption of the parasite cell membrane, leading to metabolic and structural alterations that compromise parasite survival and proliferation [32]. Germacrone and elemenone, along with germacrone-derived compounds (compounds **3** and **4**), were evaluated for antiprotozoal activity. Significantly, none exhibited cytotoxicity against Vero cells (SI > 1), indicating selective toxicity toward protozoan parasites over mammalian cells. However, germacrone exhibited cytotoxicity against canine DH82 macrophages. According to established guidelines for antiparasitic drug development, the selectivity index between parasites and host cells should ideally be at least 20 [33]. Therefore, although germacrone and its derivatives exhibit promising activity, further optimization is required to improve their activity. Germacrone demonstrated potent activity against *G. duodenalis* and *T. gallinae* trophozoites, as well as both promastigotes and amastigotes of *L. infantum*. Among the tested parasites, *L. infantum* was the most sensitive, with germacrone emerging as the compound with the broadest spectrum of antiprotozoal activity. For this reason, germacrone was selected for further studies, such as the evaluation of morphological alterations.

Germacrone has emerged as a multifunctional natural compound with a broad spectrum of biological activities. It has shown notable anticancer potential by inducing cell cycle arrest and apoptosis in various malignancies, including breast, brain, liver, skin, prostate, gastric, and esophageal cancers, primarily through the modulation of critical signaling pathways and molecular targets involved in tumor progression [34]. Beyond its anticancer effects, germacrone exhibits antiviral activity, effectively inhibiting the replication of pseudorabies virus [35] and porcine reproductive and respiratory syndrome virus [36]. Its insecticidal, acaricidal, and ixodicidal properties have also been documented [13,37] supporting its potential as a bioactive agent in pest control. Additionally, germacrone has been identified as an inhibitor of the hepatic cytochrome P450 isoform CYP2B6, suggesting possible implications for drug metabolism and pharmacokinetic interactions [38]. Furthermore, recent studies have shown that germacrone can induce oxidative stress and dysregulate cholesterol and lipid metabolism in hepatocyte-like cells, indicating a potential risk of hepatotoxicity at concentrations close to those used for therapeutic purposes [39].

Recent advances in plant biotechnology have explored innovative strategies to enhance essential oil yields. For instance, gamma irradiation combined with in vitro micropropagation of *Pelargonium graveolens* L’Hér. (1789) significantly improved biomass and EO production [40]. Similar approaches, including controlled irradiation and bioreactor-based cultivation, could be considered for *G. macrorrhizum* to increase the yield of key metabolites such as germacrone, which demonstrated the strongest antiparasitic activity in our study. Germacrone also participates in a range of chemical transformations, including transannular cyclization, epoxidation processes, and photoinduced isomerization reactions [37,41]. The cyclization and further epoxidation of germacrone lead to compounds **3** and **4** modifying the antiprotozoal activity. The diketone **3** lost the anti-*Trichomonas* and anti-*Giardia* activity whilst compound **4** lost the anti-*Giardia* activity and presented a reduction in anti-*Leishmania* activity, which became moderate. Compounds **3** and **4** have previously been evaluated for their acaricidal and insecticidal properties. Compound **3** exhibited bioactivity comparable to that of germacrone in both insecticidal and acaricidal assays. In contrast, compound 4 demonstrated repellent activity against *Rhopalosiphum padi* and moderate acaricidal effects, although its overall efficacy was lower than that of germacrone [37].

Beyond leishmanicidal activity, various sesquiterpenes have also demonstrated efficacy against other protozoan parasites. In the case of *Giardia* sp., antiparasitic effects have been reported for incomptine A analogs [42], cadinane-type sesquiterpenes [43], β-caryophyllene-4,5-α-oxide [44], brevilin A [45], and neurolenin-type furanoheliangolides such as neurolenin B [46]. Regarding *T. vaginalis*, notable activity has been observed for a chloro derivative of α-santonin [47], dihydroartemisinin [48], and caryophyllene oxide [49]. These findings underscore the broad-spectrum antiparasitic potential of sesquiterpenes and their derivatives, supporting their continued exploration as candidates for novel antiparasitic agents.

Besides the activity of germacrone on the tested protozoa metabolism, the morphological effects were also evaluated. Germacrone exerted a strong effect on the morphology of the mentioned flagellates, and the activity across *L. infantum*, *G. duodenalis*, and *T. gallinae* reveal both common and species-specific responses. In all three species, germacrone induced time-dependent alterations in cell shape and size. In *L. infantum*, germacrone caused an early increase in cell length and width, followed by a reduction in length at 48 h, indicating transient elongation and sustained cell broadening. This contrasts with *G. duodenalis*, where germacrone led to early shortening and delay widening, and with *T. gallinae*, where cell length increased only at 48 h and width decreased early. *G. duodenalis* and *T. gallinae* also showed transient nuclear narrowing followed by recovery. Germacrone promoted axoneme/axostyle elongation in both *Giardia* and *Trichomonas*, respectively, suggesting a potential stabilizing effect on microtubule-associated structures. In contrast, metronidazole consistently induced rapid and sustained morphological damage in both protozoa, including axostyle/axoneme loss and nuclear shrinkage. These findings suggest that germacrone elicits a more dynamic and potentially reversible response across different protozoan parasites, whereas metronidazole exerts a more immediate and degenerative effect. The differential responses observed highlight the importance of parasite-specific structural features in shaping drug sensitivity and may inform the development of broad-spectrum antiparasitic strategies.

The specific mode of action of germacrone against protozoa has not been detailed so far, but other natural terpenes with anti-*Giardia* activity have different mechanisms of action. Andrographolide, a diterpenoid lactone, caused significant alterations in *G. duodenalis* trophozoite shape and size and effectively inhibited trophozoite adhesion [50]. Other molecules, such as linearolactone, a neo-clerodane-type diterpene, induced partial arrest in the S phase of the trophozoite cell cycle without evidence of reactive oxygen species (ROS) production. It also triggered pronecrotic cell death and ultrastructural alterations, including changes in vacuole abundance, the appearance of perinuclear and periplasmic spaces, and glycogen granule deposition [51]. Additionally, other terpenes like the sesquiterpene lactone dihydroartemisinin with activity against related protozoa, such as *T. vaginalis*, it disrupts membrane systems, further illustrating the mechanistic diversity of this compound class [48].

Several sesquiterpene lactones have demonstrated antileishmanial activity through diverse mechanisms. Helenalin, mexicanin, and dehydroleucodine inhibited the in vitro growth of *Leishmania* promastigotes, with evidence of DNA fragmentation, and in the case of helenalin, pronounced vacuolization was also observed [52]. Similarly, (-)-α-bisabolol showed efficacy against intracellular amastigotes of *L. tropica*, inducing oxidative stress and mitochondrial-dependent apoptosis without compromising plasma membrane integrity [53]. In contrast, γ-elemene exhibited direct activity against both promastigote and amastigote forms of *L. amazonensis*, primarily by disrupting plasma membrane integrity [54]. These findings highlight the mechanistic diversity of sesquiterpene lactones and their potential as antiparasitic agents targeting different cellular pathways in *Leishmania*. Altogether, it seems that terpenes and sesquiterpenes exert a remarkable effect on both, the metabolic activity of the protozoa and the protozoa architecture, as we have observed with germacrone in the present study.

## 4. Materials and Methods

### 4.1. Plant Material

Pre-selected field-grown plants from a *G. macrorrhizum* cultivar (voucher number R297730), previously reported [13] for superior biomass production, were collected from Ejea de los Caballeros (Spain) and subsequently propagated under greenhouse conditions. All rhizomes obtained were propagated and transferred to a VENLO-type greenhouse under controlled conditions (24 °C, 70% relative humidity). Rhizomes were cultivated in 1 L containers filled with Compo Universal substrate, to which 10% (*v*/*v*) perlite was added to improve aeration and drainage. After two months, plants were transplanted into individual 5 L pots filled with commercial substrate. Plants were irrigated three times per week, or as needed, during spring, autumn, and winter. Weed control was carried out manually on a weekly basis. Irrigation was provided by a micro-sprinkler system (0.7 L h^−1^ per event), supplying approximately 0.12 L per pot per week. Fertilization was applied both in solid and liquid forms. Solid fertilization was carried out using “Compo Novatec” fertilizer with micronutrients, at a dose of 3 g per pot every six months. Liquid fertilization was conducted monthly using “Compo Huerto y Frutales” (COMPO GmbH, Münster, Germany) (rich in potassium), following the manufacturer’s recommended dosage. The plants flowered normally throughout this period.

### 4.2. Essential Oils

The essential oils (EOs) were obtained from the plant material by hydrodistillation (Clevenger) from flowers (EOF) and aerial parts (leaves and stems) (EOAP) with yields of 0.32% for EOF and 0.56% for EOAP, respectively. A 100 g sample of *G. macrorrhizum* aerial parts was hydrodistilled in a Clevenger-type apparatus for 2 h; the resulting EO was dried over anhydrous ammonium sulfate, filtered, and stored at 4 °C for subsequent utilization.

### 4.3. Analysis

EOs were analyzed by gas chromatography–mass spectrometry (GC-MS) using a Shimadzu GC-2010 Plus system coupled to a GCMS-QP2010-Ultra detector (EI, 70 eV, Single Quadrupole; Shimazdu, Kyoto, Japan) with helium as carrier gas following the methodology described by Bailén et al. [55]. Separation was performed on a TRB-5 capillary column (30 m × 0.25 mm, 0.25 μm film thickness). Injection was in split mode (20:1) with 1 μL of sample dissolved in dichloromethane (4 μg/μL). The oven program started at 70 °C, increased to 290 °C at 6 °C/min, and held for 15 min. Injector, transfer line, and ion source temperatures were 300 °C, 250 °C, and 220 °C, respectively. Compound identification was based on retention times and mass spectra compared with Wiley and NIST libraries, and quantification was performed using relative peak area percentages.

### 4.4. Compounds

**Figure 7:**
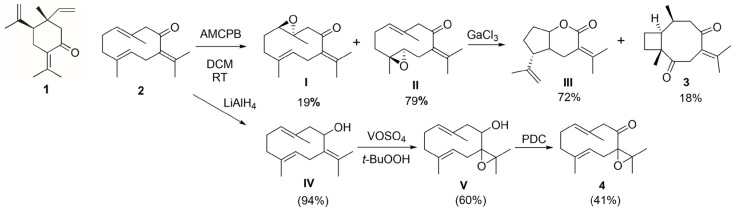
Chemical structures of β-elemenone (**1**), germacrone (**2**) and synthesis pathways leading to compounds **3** and **4** derived from germacrone.

Compounds selected for further study included the major constituents: β-elemenone (**1**), germacrone (**2**), previously isolated from the plant, and compounds **3** and **4** generated from germacrone (**2**), following protocols already reported by Galisteo et al. [37] (Figure 7). Diketone (**3**) was produced after Ga(III)-mediated tandem cyclization-rearrangement of the 4-5-epoxide of germacrone, whereas the generation of 4 involved the reduction of germacrone with LAH followed by the hydroxyl-directed epoxidation of IV to form epoxide V, which was oxidized with PDC to produce 4.

### 4.5. Evaluation of Antiparasitic Effects of G. macrorrhizum Extracts and Main Components on Extracellular Protozoa

Anti-*Giardia* (AG) activity assays were conducted on *G. duodenalis* trophozoites from the ATCC^®^ 30957 strain (assemblage A), using flat-bottom microwell plates in quadruplicate. Each well contained 150 μL of *G. duodenalis* trophozoites suspended in modified TYI-S-33 medium [56], supplemented with 10% heat-inactivated fetal bovine serum (FBS) (Sigma, Madrid, Spain) at a concentration of 10^6^ trophozoites/mL. Incubation was carried out for 24 h at 37 °C.

The anti-*Trichomonas* (AT) activity assay utilized *T. gallinae* trophozoites from an isolate obtained from a wood pigeon by Dr. Mª Teresa Gómez, employing round-bottom microwell plates in quadruplicate. Each well was filled with 150 μL of *T. gallinae* trophozoites in Trypticase-Yeast Extract-Maltose (TYM) medium, supplemented with 10% FBS at a concentration of 500,000 trophozoites/mL, with incubation for 24 h at 37 °C.

Anti-*Leishmania* (AL) activity was assessed on *L. infantum* promastigotes (MCAN/ES/98/LLM-722), kindly provided by Dr. J.M. Requena from CBM-CSIC, as well as on *L. infantum* (M/CAN/ES/96/BCN150 zymodeme MON-1), strain isolated from a dog with active visceral leishmaniasis [57]. *L. infantum* promastigotes (MCAN/ES/98/LLM-722) were cultured in RPMI medium supplemented with 15% FBS and 10 μg/mL of hemin (Acros Organics, Madrid, Spain), grown at 26 °C. Promastigotes in logarithmic growth were seeded in 96-well flat-bottom plates at a volume of 100 μL of culture per well and incubated for 48 h. Promastigotes for macrophage infection (M/CAN/ES/96/BCN150 zymodeme MON-1) were maintained at 26 °C in Schneider’s insect medium (Lonza, Basel, Switzerland) supplemented with 10% FBS and 100 U/mL penicillin and 100 μg/mL streptomycin (Lonza, Basel, Switzerland) (Pen-Strep). Promastigotes in the stationary phase of growth (5–6 days) were washed twice in phosphate-buffered saline at 2700× *g* for 10 min at 22 °C and employed for macrophage infection.

EOs and pure compounds were solved in DMSO (Sigma, Madrid, Spain) (<1% final concentration). The EOs and extracts were tested under concentrations of 800, 400, and 200 μg/mL, and in those cases that showed moderate antiparasitic activity (>50%) at the lowest concentration, additional testing was performed at lower concentrations: 100, and 50 μg/mL. Trophozoites/promastigotes viability was assessed using the modified MTT colorimetric assay, as described previously in the literature [23,55,58], with results further validated by microscopic examination. The plate was analyzed in a spectrophotometer at a wavelength of 570 nm. The percentage of antiparasitic activity was calculated as growth inhibition, using the following formula: % AT = 100 − [(Ap − Ab) ÷ (Ac − Ab)] × 100, where Ap is the absorbance of the tested product, Ab the absorbance of the blank and Ac the absorbance of the control wells (culture without treatment).

Pure compounds were tested using the same method, although at concentrations of 100, 75, 50, 25, 10, and 1 μg/mL. Metronidazole (Acros Organics, Madrid, Spain) served as a reference compound for anti-*Giardia* activity and Anti-*Trichomonas* activity and amphotericin B for anti-*Leishmania* activity.

### 4.6. Evaluation of Antileishmanial Effects of Germacrone on Intracellular Amastigotes

#### 4.6.1. Macrophages

Canine DH82 (ATCC^®^-CRL-10389TM) macrophages were maintained in Dulbecco’s modified Eagle’s medium (DMEM) (Thermo Fisher Scientific, Waltham, MA, USA) supplemented with 10% heat-inactivated FBS (Thermo Fisher Scientific, Waltham, MA, USA), and Pen-Strep at 37 °C in 5% CO_2_.

#### 4.6.2. Cytotoxicity of Germacrone on Mammalian Cells

DH82 canine macrophages (5 × 10^4^ per well) were seeded in 96-well plates in DMEM and kept overnight at 37 °C and 5% CO_2_. After washing with PBS, germacrone was added in varying concentrations (10, 50 and 100 μg/mL). After 48 h of treatment, cell viability was determined by the MTT method using a spectrophotometer (Multiskan EX, Thermo Electron Corporation, Vantaa, Finland) at 570 nm. The results were expressed as the cytotoxic concentration of 50% of the cell population (CC_50_). Amphotericin B (Gibco, Thermo Fisher Scientific, Grand Island, NY, USA) at 1 μg/mL was used as the reference drug. The assays were performed in two independent experiments, each in quadruplicate.

#### 4.6.3. Infection Index of Macrophages

DH82 cells (5 × 10^4^ per well) were seeded in LabTek culture chamber slides (Thermo Scientific, Nunc™, Roskilde, Denmark) and incubated overnight at 37 °C and 5% CO_2_. On the following day, macrophages were infected with *L. infantum* stationary phase-promastigotes at a ratio of 5:1 parasites/macrophage and after 4 h of incubation at 37 °C, a time point that reflects initial infection, extracellular promastigotes were removed by washing. Germacrone was added at varying concentrations (10, 50 and 100 μg/mL) and macrophages were incubated in fresh medium for 48 h at 37 °C and 5% CO_2_. After Giemsa staining, cells were mounted with Vitro-Clud (Deltalab SLU, Barcelona, Spain), and 400 macrophages were counted in duplicate in a microscope Olympus BX41 (Olympus Corporation, Tokyo, Japan). The percentage of infected macrophages and the mean of the number of amastigotes per infected macrophage (defined as the intensity of infection) were evaluated. The infection index was calculated by multiplication of both parameters to account for the overall parasite load [59].

### 4.7. Cytotoxicity of Pure Compounds

African green monkey kidney cells (Vero cells) were maintained in Dulbecco’s modified Eagle’s minimal essential medium (DMEM) (Thermo Fisher Scientific, Waltham, MA, USA) supplemented with 10% FBS and 1% Pen-Strep at 37 °C under a humidified atmosphere of 5% CO_2_/95% air.

Cells seeded in 96-well flat-bottom microplates with 100 μL medium per well (initial densities 10^4^ cells per well) were exposed for 48 h to serial dilutions (100, 75, 50, 25, 10 and 1 µg/mL) of the tested compounds in DMSO (<1% final concentration). Cell viability was analyzed by the MTT colorimetric assay method, and the purple-colored formazan precipitate was dissolved with 100 μL of DMSO [23].

### 4.8. Morphological Evaluation of the Anti-Protozoan Effects

*L. infantum*, *T. gallinae* and *G. duodenalis* were studied at 0, and 48 h after treatment with germacrone (100 µg/mL), and controls (DMSO 1%, metronidazole at 10 µg/mL for *T. gallinae* and *G. duodenalis* and amphotericin B at 1 µg/mL for *L. infantum*).

For *L. infantum* and *T. gallinae*, cultures in the exponential growth phase (10^7^ promastigotes/mL and 5 × 10^5^ trophozoites/mL, respectively) were treated in 96-well microplates. At each time point, quadruplicates were assigned per condition: growth control, germacrone, and the corresponding positive control (metronidazole or amphotericin B). For microscopy, quadruplicate wells corresponding to the same condition/time point were pooled prior to centrifugation to obtain sufficient biomass and a representative pellet for smear preparation. Pooled samples per condition/time point were centrifuged, resuspended in FBS and spread onto microscope slides. Once dried, samples were fixed and stained with Diff-Quik (Medion Diagnostic AG, Düdingen, Switzerland) following the manufacturer’s instructions. Additionally, three wells per condition (triplicates) were used for the MTT assay, with absorbance measured by spectrophotometry after 48 h.

For *G. duodenalis*, Lab-Tek chamber slides (8 wells) were prepared with Keister culture medium and inoculated with trophozoites (10^6^ cells/mL). Each well contained 435 µL of culture medium and 15 µL of the test or control compounds at the above-mentioned concentrations. The chamber included: two wells for growth control (0.5 h, 48 h), two for germacrone (0.5 h, 48 h), and two for metronidazole (0.5 h, 48 h). Incubation was performed at 37 °C. At each time point, the medium was removed, wells were dried, fixed, and stained with Diff-Quik.

Morphological changes were analyzed using light microscopy. Experiments were performed as independent biological replicates on different plates, and one Diff-Quik stained smear was obtained per condition and time point per biological replicate. For morphometric analysis, a total of 30 trophozoites per condition and time point were measured across the independent biological replicates and were selected by consecutive sampling during a predefined serpentine (zig-zag) scan across the preparation. Up to two eligible trophozoites per field (intact, well-focused and non-overlapping) were measured in sequential fields until *n* = 30 was reached, avoiding subjective selection of “representative” cells. Overlapping, partially visible, out-of-focus, edge-of-field, or debris-obscured trophozoites were excluded a priori. Trophozoites were photographed using a researcher’s mobile phone mounted to the microscope ocular. Calibration was performed at the same magnification using a stage micrometre and applied in ImageJ v. 1.54d. One Diff-Quik stained smear was obtained per condition/time point per biological replicate Cell length, cell width, cell surface, nuclear length, nuclear width, axoneme (only *Giardia*), and length of axostyle (only *T. gallinae*) were measured with ImageJ software, and the data were recorded in an Excel database for statistical analysis.

### 4.9. Statistical Analysis

Data was analyzed using STATGRAPHICS Centurion XIX (https://www.statgraphics.com) (Statgraphics Technologies, Inc., The Plains, VA, USA). The mammalian cells and trophozoites viability were tested with each compound in a dose–response experiment to calculate their relative potency (CC_50_ or IC_50_ value, respectively). IC_50_ (μg/mL) expresses the dose of EOs, extracts or pure compounds needed to produce 50% mortality of trophozoites, while CC_50_ (μg/mL) expresses the dose of compounds necessary to produce 50% mortality of Vero cells.

Selectivity index was calculated for the antiparasitic activity of pure compounds, using the formula SI = CC_50_/IC_50_. Compounds with SI higher than one were considered as potential anti-parasitic compounds, since they are more toxic for protozoans than for mammalian cells.

The Shapiro–Wilk test was used to assess the normality of the morphological variables. When data followed a normal distribution, Student’s *t*-test was applied; otherwise, non-parametric tests, such as the Mann–Whitney U test, was used. Values of *p* < 0.05 were considered significant. For the morphological analysis, the mean, standard deviation, median, and interquartile range were calculated using SPSS version 20 (IBM Corp., Armonk, NY, USA).

## 5. Conclusions

The results of this study demonstrate that the essential oils (EOs) from *G. macrorrhizum*, which are rich in germacrone, exhibit significant antiparasitic activity against *G. duodenalis*, *T. gallinae*, and *L. infantum*. Germacrone emerges as a key bioactive constituent, showing broad-spectrum efficacy and selective toxicity toward protozoan parasites (SI > 1). These properties support its potential as a lead compound for the development of novel antiparasitic therapies. Consequently, *G. macrorrhizum* represents a promising natural source of antiprotozoal agents, warranting further investigation into its active components and underlying mechanisms of action.

## Figures and Tables

**Table 1:** Effects of EOs from *G. macrorrhizum* on *L. infantum* promastigotes and *G. duodenalis* and *T. gallinae* trophozoites (IC_50_).

*G. macrorrhizum*		*G. duodenalis* ^a^		*L. infantum* ^a^		*T. gallinae* ^a^	
		IC_50_	R^2^	IC_50_	R^2^	IC_50_	R^2^
EOs	EOAP	>500	-	69.6 (62.5–77.4)	91.3	120.4 (108.0–134.3)	94.7
	EOF	>300	-	>800	-	67.2 (63.1–71.6)	97.1

Data from two independent experiments are shown, each performed in quadruplicate wells (technical replicates). ^a^ IC_50_ (μg/mL) = concentration needed to produce 50% trophozoite mortality; IC_50_ values were calculated from dose–response curves using viability assays. Control: DMSO 1%; solvent: DMSO 1%.

**Table 2:** Chemical composition of extracts obtained through sequential extraction of EOs from *G. macrorrhizum* cultivated in a greenhouse.

RT	RI	% Relative Abundance		Compound	Class	% Relative Abundance	
		EOAP	EOF			EOAP	EOF
13.89	1369	3.05	1.11	g-elemene	Non oxygenated sesquiterpenes	6.77	1.11
14.92	1485	3.72	-	(+)-cuparene			
17.49	1609	32.64	42.07	β-elemenone (**1**)	Oxygenated sesquiterpenes	73.4	78.9
18.53		3.74	3.23	70/121/93/42/67/107/41/95/79/81	-		
19.36	1704	40.72	36.85	germacrone (**2**)	Oxygenated sesquiterpenes		

RI: retention index; RT: retention time.

**Table 3:** Antiparasitic effects of isolated compounds of *G. macrorrhizum* (IC_50_ (µg/mL) against *L. infantum* promastigotes and *G. duodenalis* and *T. gallinae* trophozoites.

Compound	Vero Cells	*L. infantum* (pm)			*G. duodenalis*			*T. gallinae*		
	CC_50_ (µg/mL) ^a^	IC_50_ (µg/mL) ^b^	R^2^	SI ^c^	IC_50_ (µg/mL) ^b^	R^2^	SI ^c^	IC_50_ (µg/mL) ^b^	R^2^	SI ^c^
**1** (β-elemenone)	>100	>100	-	1.0	>100	-	1	>100	-	1.0
**2** (germacrone)	≈100	37.5 (33.6–41.9)	91.1	2.7	55.2 (48.2–63.2)	77.6	1.8	45.8 (37.2–56.3)	77.4	2.2
**3**	>100	35.5 (31.2–40.4)	92.1	2.8	>100	-	1	>100	-	1.0
**4**	>100	88.8 (62.6–125.9)	78.0	1.1	>100	-	1	51.6 (48.6–54.8)	96.2	1.9
AmB	7.11 [21]	0.01 [22]	-	711	-	-	-	-	-	-
Met	>100	-	-	-	4.4 (3.5–5.5)	96.1	22.7	1.0 (0.8–1.1) [23]	-	100

Data from two independent experiments are shown each performed in quadruplicate wells (technical replicates). ^a^ CC_50_ (μg/mL) = concentration needed to produce 50% Vero cell mortality; ^b^ IC_50_ (μg/mL) = concentration needed to produce 50% trophozoite mortality; ^c^ SI: Selectivity index (CC_50_/IC_50_); pm = promastigotes. AmB: amphotericin B; Met: metronidazole; control: DMSO 1%; solvent: DMSO 1%. IC_50_ and CC_50_ values were calculated from dose–response curves using viability assays.

**Table 4:** In vitro activity of germacrone against intracellular amastigotes of *L. infantum*.

	% Infected Cells	nº Amastigotes/Infected Cell	Infection Index
control of infection	42.8 ± 6.8	3.5 ± 1.8	152.1 ± 29.4
DMSO 1%	46.9 ± 6.9	2.7 ± 1.4	126.6 ± 4.9
AmB (1 µg/mL)	6.4 ± 3.7 *	1.4 ± 0.7 *	8.9 ± 0.0 *
Germacrone (**2**) 10 µg/mL	25.9 ± 8.3 *	2.4 ± 1.1	61.0 ± 9.4 *
Germacrone (**2**) 50 µg/mL	dead cells	dead cells	dead cells
Germacrone (**2**) 100 µg/mL	dead cells	dead cells	dead cells

Data (mean ± SD) from two independent experiments are shown (technical replicates). (* *p* < 0.05, Student’s *t* test). AmB: amphotericin B; solvent DMSO 1%; control: no treatment.

## Data Availability

All data supporting the findings of this study are available within the article.

## Abbreviations

The following abbreviations are used in this manuscript:

AG	anti-*Giardia*
AL	anti-*Leishmania*
AmB	Amphotericin B
AT	anti-*Trichomonas*
CC_50_	50% cytotoxicity concentration
DMEM	Dulbecco’s modified Eagle’s medium
EOs	Essential Oils
EOAP	Essential Oils from aerial parts
EOF	Essential Oils from flowers
FBS	fetal bovine serum
GC-MS	Gas Chromatography–mass spectrometry
IC_50_	Half Maximal Inhibitory Concentration
Met	Metronidazoles
PBS	phosphate-buffered saline
